# Vaccination coverage survey by social stratum in children up to 24 months of age in Londrina, Paraná, Brazil, between 2021 and 2022

**DOI:** 10.1590/S2237-96222024v33e20231393.especial2.en

**Published:** 2024-10-21

**Authors:** Edmilson de Oliveira, José Cássio de Moraes, Ana Paula França, ICV 2020 Group, Adriana Ilha da Silva, Alberto Novaes Ramos, Ana Paula França, Andrea de Nazaré Marvão Oliveira, Antonio Fernando Boing, Carla Magda Allan Santos Domingues, Consuelo Silva de Oliveira, Ethel Leonor Noia Maciel, Ione Aquemi Guibu, Isabelle Ribeiro Barbosa Mirabal, Jaqueline Caracas Barbosa, Jaqueline Costa Lima, José Cássio de Moraes, Karin Regina Luhm, Karlla Antonieta Amorim Caetano, Luisa Helena de Oliveira Lima, Maria Bernadete de Cerqueira Antunes, Maria da Gloria Teixeira, Maria Denise de Castro Teixeira, Maria Fernanda de Sousa Oliveira Borges, Rejane Christine de Sousa Queiroz, Ricardo Queiroz Gurgel, Rita Barradas Barata, Roberta Nogueira Calandrini de Azevedo, Sandra Maria do Valle Leone de Oliveira, Sheila Araújo Teles, Silvana Granado Nogueira da Gama, Sotero Serrate Mengue, Taynãna César Simões, Valdir Nascimento, Wildo Navegantes de Araújo

**Affiliations:** 1 Santa Casa de São Paulo, Faculdade de Ciências Médicas, São Paulo, SP, Brazil; Universidade Federal do Espírito Santo, Vitória, ES, Brazil; Universidade Federal do Ceará, Departamento de Saúde Comunitária, Fortaleza, CE, Brazil; Faculdade Ciências Médicas Santa Casa de São Paulo, São Paulo, SP, Brazil; Secretaria de Estado da Saúde do Amapá, Macapá, AP, Brazil; Universidade Federal de Santa Catarina, SC, Brazil; Organização Pan-Americana da Saúde, Brasília, DF, Brazil; Instituto Evandro Chagas, Belém, PA, Brazil; Faculdade de Ciências Médicas Santa Casa de São Paulo, Departamento de Saúde Coletiva, São Paulo, SP, Brazil; Universidade Federal do Rio Grande do Norte, Natal, RN, Brazil; Universidade Federal do Ceará, Departamento de Saúde Comunitária, Fortaleza, CE, Brazil; Universidade Federal de Mato Grosso, Cuiabá, MT, Brazil; Universidade Federal do Paraná, Curitiba, PR, Brazil; Universidade Federal de Goiás, Goiânia, GO, Brazil; Universidade Federal do Piauí, Teresina, PI, Brazil; Universidade de Pernambuco, Faculdade de Ciências Médicas, Pernambuco, PE, Brazil; Instituto de Saúde Coletiva, Universidade Federal da Bahia, Salvador, BA, Brazil; Secretaria de Estado da Saúde de Alagoas, Maceió, AL, Brazil; Universidade Federal do Acre, Rio Branco, AC, Brazil; Universidade Federal do Maranhão, Departamento de Saúde Pública, São Luís, MA, Brazil; Universidade Federal de Sergipe, Aracaju, SE, Brazil; Secretaria Municipal de Saúde, Boa Vista, RR, Brazil; Fundação Oswaldo Cruz, Mato Grosso do Sul, Campo Grande, MS, Brazil; Fundação Oswaldo Cruz, Escola Nacional de Saúde Pública Sergio Arouca, Rio de Janeiro, RJ, Brazil; Universidade Federal do Rio Grande do Sul, Porto Alegre, RS, Brazil; Fundação Oswaldo Cruz, Instituto de Pesquisa René Rachou, Belo Horizonte, MG, Brazil; Secretaria de Desenvolvimento Ambiental de Rondônia, Porto Velho, RO, Brazil; Universidade de Brasília, Brasília, DF, Brazil

**Keywords:** Cobertura Vacunal, Esquema de Inmunización, Encuestas Epidemiológicas, Evaluación de Programa, Vaccination Coverage, Immunization Schedule, Epidemiological Surveys, Program Evaluation

## Abstract

**Objective:**

To analyze vaccination coverage according to social strata in children up to 24 months old, living in the municipality of Londrina (PR), Brazil.

**Methods:**

This was a population-based survey conducted between 2021 and 2022, in which vaccination coverage and sociodemographic aspects of mothers and families were evaluated using Pearson’s chi-square test.

**Results:**

In a sample of 456 children, complete vaccination coverage varied according to social strata, being 36.0% (95%CI 26.8;57.8); in stratum A; 59.5% (95%CI 26.1;86); in stratum B; 66.2% (95%CI 51.7;78.1); in stratum C; and 70.0% (95%CI 56.1;81.0) in stratum D.

**Conclusion:**

The analysis of vaccination coverage indicated that social stratum A is at highest risk for vaccine-preventable diseases.

## Study contributions

**Main results:**

The results of the study showed low full vaccination coverage in children up to 24 months of age in Londrina, being higher in the less financially advantaged social stratum, compared to the most advantaged.

**Implications for services:**

The results found can support the qualification of the immunization program and enable, based on planning and ongoing health education, the definition of unique strategies to improve vaccination coverage.

**Perspectives:**

Future perspectives point to the importance of carrying out investigations into the challenges inherent to vaccination, as well as qualitative and quantitative research addressing health professionals to better understand the data.

## RESUMEN

**Objetivo:**

Análisis de la cobertura de vacunación según el estrato social, en niños de hasta 24 meses de vida, residentes en el municipio de Londrina (PR)

**Métodos:**

Encuesta de base poblacional, realizada entre 2021 y 2022, en la que se evaluó la cobertura de vacunación y los aspectos sociodemográficos de las madres y las familias, a través de la prueba chi-cuadrado de Pearson.

**Resultados:**

En una muestra de 456 niños, la cobertura de vacunación completa varió según el estrato social, siendo en el estrato A del 36,0% (IC_95_% 26,8;57,8), en el estrato B del 59,5% (IC_95_% 26,1;86), en el estrato C del 66,2% (IC_95_% 51,7;78,1) y en el estrato D del 70,0% (IC_95_% 56,1;81,0).

**Conclusión:**

El análisis de la cobertura de vacunación indicó que el estrato social A es el de mayor riesgo para enfermedades prevenibles por vacunación.

## INTRODUCTION

Accurate knowledge of vaccination coverage in children is one of the important elements for evaluating epidemiological surveillance programs, as it allows monitoring the increase in the number of susceptible individuals in the population, as well as evaluating the effectiveness of acquired immunity as a barrier to transmission of vaccine-preventable diseases.^1^

Vaccines are important investments in public health as they prevent infectious diseases, reduce the severity of diseases and support the reduction of child mortality, presenting high efficacy, safety and cost-benefit.^2^ In the last 20 years, there has been great commitment from health managers aimed at the global advancement of vaccination programs, which have been successful in poor countries, with more than 300 million children immunized and around 4 million children saved.^3^

In Brazil, the National Immunization Program (*Programa Nacional de Imunizações* - PNI) is the body responsible for promoting the control of communicable diseases through routine vaccination, periodic vaccination campaigns and epidemiological surveillance of communicable diseases.^4^ Sustaining vaccination coverage at recommended levels to ensure children’s health continues to be a challenge,^5^ with recommended targets of 90% for the Bacillus Calmette-Guérin (BCG) and human rotavirus vaccines; and 95% for vaccines against poliomyelitis; diphtheria, tetanus and pertussis (DTP); measles, mumps and rubella (MMR); hepatitis B; yellow fever; 5-in-1; meningococcal C conjugate vaccine; pneumococcal 10-valent vaccine; varicella and hepatitis A vaccines and tetravalent vaccine.^4^

The last national household vaccination coverage survey, carried out in 2007 in Brazilian state capitals and Federal District, showed the following coverage percentages: 97% for BCG, 96% for poliomyelitis, 94% for DTP, 91% for hepatitis B and 91% for MMR, indicating that only the BCG and poliomyelitis vaccines reached the recommended parameters.^4,6^ As for the full valid-dose vaccination schedule, i.e. doses administered within the period recommended by the PNI, the study identified overall coverage of 68.0%.^7^

In addition to investigating vaccination coverage, studies have sought to understand how social determinants affect vaccination among children. In Brazil, between 2010 and 2020, lower vaccination coverage and greater downward trends were related to lower Human Development Indexes, concentrated in the North and Northeast regions of the country.^8^

Vaccination coverage survey-type studies are essential tools for addressing these disparities, as they make it possible to demonstrate vaccination status reliably; identify the number of people susceptible to infectious diseases; help understand access and adherence to the PNI; support analysis and reliability of administrative data; as well as investigating living conditions that interfere with vaccination coverage, seeking to identify social inequalities in health.^7^

Considering the importance of maintaining good vaccination coverage in order to promote the health of children up to 24 months of age and the need to know the vaccination situation according to social status in order to understand the current challenges to be addressed, the present study aimed to analyze vaccination coverage according to social strata among children up to 24 months old, living in the city of Londrina (PR), Brazil.

## METHODS

This is a household-based survey study that identified vaccination coverage in children up to 24 months old, conducted from October 2021 to January 2022. This study is part of an expanded investigation called “Survey of Vaccination Coverage in municipalities with over 200,000 inhabitants” (“*Inquérito de Cobertura Vacinal em municípios com mais de 200.000 habitantes*”).

This study was conducted in the municipality of Londrina, located in the northern region of the state of Paraná, Brazil, which in 2020 had a resident population of 575,377 inhabitants and population density of 306.52 inhabitants/km.^9^ Primary Health Care in Londrina includes 54 Primary Health Centers and 74 vaccination rooms.^10^

The inclusion criterion referred to children born alive in 2017 and 2018 and living in the urban area of the city of Londrina. The population base was obtained from the Live Birth Information System (*Sistema de Informações sobre Nascidos Vivos* - SINASC) and the follow-up period was retrospective. Newborns were georeferenced according to their census tracts of residence,^11^ obtained via the 2010 Demographic Census.^9^

The sample size took into account the design effect due to the use of clusters of census tracts, established at 1.4, based on previous studies; a hypothetical population of 14,000 live births; estimated 70% vaccination coverage prevalence, estimation error of 5% and 1.96 for a 95% confidence interval.^12^ The sample was spread over four social strata in the census tracts, consisting of approximately 113 children. In addition to using disproportionate allocation procedures, it was necessary to calculate and use sampling weights for each household interviewed, in order to enable unbiased estimation of the parameters of interest in the population.^12^

The data used to classify the census tracts were: average income of heads of household, proportion of literate heads of household and proportion of heads of household with income greater than or equal to 20 minimum wages. They were then divided into social strata A (high), B (medium-high), C (medium-low) and D (low). The tracts were grouped into conglomerates by means of cluster analysis.^12^

In order to identify level of family consumption, families were classified according to the criteria of the Brazilian Association of Survey Companies (*Associação Brasileira de Empresas de Pesquisa* - ABEP),^13^ as: A, B, C and D, where A was the highest level of consumption and D was the lowest level of consumption. The household characteristics considered were: number of people living in the household, number of rooms serving as bedrooms, number of bathrooms for exclusive use by residents, number of passenger cars for exclusive family use, number of motorcycles for exclusive use, number of monthly employees, number of refrigerators, freezers, washing machines, dishwashers, microwave ovens, clothes dryers, DVD players and microcomputers.^12^

Data collection was performed by a company specialized in carrying out household surveys, following the legal requirements for hiring its services, ensuring that the field researchers had training in the area of health, experience in data collection and knowledge about immunization. The variables were obtained by administering a questionnaire and information on vaccine doses administered was obtained by taking legible photographs of each child’s vaccination card(s). These data were read by health professionals who work with vaccination and input to the electronic questionnaire. 

Regarding the children’s characteristics, the variables assessed were: sex (male and female) and self-reported race/skin color (White, Black, mixed race, Asian and Indigenous). The mothers’ characteristics were: maternal age group (< 20 years, 20-34 years and over 35 years), schooling (up to 8 years, 9-12 years, 13-15 years and 16 years or more), self-reported race/skin color (White, Black, mixed race, Asian and Indigenous) and receiving family benefit (*Bolsa* *Família*) (yes). Monthly family income was taken as: up to BRL 300; BRL 301 to BRL 1,000; BRL 1,001 to BRL 3,000; BRL 3,001 to BRL 5,000. When evaluating consumption levels separately, only a small proportion was identified at level A, so that we opted to group levels A and B together; and C and D together.

The vaccines included in the study were: BCG, hepatitis B, rotavirus, 5-in-1, IPV (inactivated poliovirus vaccine), pneumococcal, meningococcal C, OPV (oral poliovirus vaccine), hepatitis A, DTP (diphtheria, tetanus and pertussis) and MMR (measles, mumps and rubella). The National Immunization Program guidelines for the full vaccination schedule at 24 months old were taken as the standard, which define the vaccination schedule as being complete when at least 90% of children have been vaccinated against BCG (one dose) and human rotavirus (two doses), and 95% have been vaccinated against poliomyelitis (three doses and a booster), DTP (three doses and a booster), MMR (two doses), hepatitis B (one dose), yellow fever (one dose), 5-in-1 (three doses), meningococcal C conjugate (three doses), pneumococcal 10-valent (three doses), varicella (one dose), hepatitis A (one dose) and tetravalent (one dose).^4^

Vaccination coverage was calculated taking the number of vaccines administered as the numerator and the number of children up to 24 months old in the city of Londrina as the denominator. The proportion of children vaccinated in each social stratum was also calculated, thus identifying coverage in each stratum analyzed.

All the analyses were performed with Stata® version 17, using its survey analysis module, considering the sample weights and the study design. In order to identify statistical differences, vaccination coverage was presented with its respective confidence intervals (95%CI), and differences in the proportions of variables between groups of children were assessed using Pearson’s chi-square test with 5% significance (p < 0.05).

Study participants signed a Free and Informed Consent Form, and data confidentiality was ensured by the researchers involved. The study was approved by the Human Research Ethics Committee of the *Instituto de Saúde Coletiva da Universidade Federal da Bahia*, as per Opinion No. 3.366.818 dated June 4, 2019, and as per Certificate of Submission for Ethical Appraisal No. 4306919.5.0000.5030, and by the Human Research Ethics Committee of the *Irmandade da Santa Casa de São Paulo*, as per Opinion No. 4.380.019, dated November 4 , 2020, and as per Certificate of Submission for Ethical Appraisal No. 39412020.0.0000.5479. 

## RESULTS

A total of 456 children participated in the study, obtained from a population of 14,091 live births in 2017 and 2018 in the city of Londrina. The study included 3.1% of this population, with no losses. Of the total number of children in the sample, 56.0% were male and 44.0% were female. With regard to race/skin color, 65.0% were reported as being White; 30.0% mixed race; 3.0% Black; 1.0% Indigenous; and 1.0% Asian (Table 1).

**Table 1:** Sociodemographic characteristics of children up to 24 months old, by social strata, Londrina, Paraná, Brazil, 2021-2022 (n = 456)

**Characteristics**	**Social strata (%)**					
	**A (115)**	**B (114)**	**C (113)**	**D (114)**	**Total (456)**	**p-value** ^a^
**Sex**						< 0.205
Male	57.7	53.4	62.3	48.0	56.0	
Female	42.3	46.6	37.7	52.0	44.0	
**Race/skin color**						< 0.388
White	89.0	61.0	77.0	43.5	65.0	
Black	0.0	0.0	8.0	4.0	3.0	
Mixed race	6.0	38.0	15.0	52.5	30.0	
Asian	5.0	0.0	0.0	0.0	1.0	
Indigenous	0.0	1.0	0.0	0.0	1.0	

a) Chi-square test.

The most prevalent maternal age group was 20 to 34 years old, accounting for 29.0% of mothers in stratum A, 44.4% in stratum B, 71.9% in stratum C and 76.1% in stratum D. Mothers under 20 years old were found in a higher proportion in stratum B (21.8%). The group of mothers aged 35 or over was more prevalent in stratum A, accounting for 70.0% of the total (Table 2). 

**Table 2:** Maternal and family characteristics of children up to 24 months old, by social strata, Londrina, Paraná, Brazil, 2021-2022 (n = 456)

**Characteristics**	**Social strata (%)**				
	**A (n = 115)**	**B (n = 114)**	**C (n = 113)**	**D (n = 114)**	**p-value** ^a^
**Maternal age group (in years)**					< 0.001
Under 20	1.0	21.8	2.2	2.4	
20-34	29.0	44.4	71.9	76.1	
35 or over	70.0	33.8	25.9	21.5	
**Schooling (in years)**					< 0.003
Up to 8	1.0	33.1	15.6	36.2	
9-12	2.4	2.0	16.5	22.4	
13-15	13.6	25.1	49.0	30.3	
16 or over	82.3	39.3	17.9	9.3	
**Race/skin color**					< 0.032
White	85.8	57.9	70.0	39.0	
Black	2.4	1.1	11.0	12.0	
Mixed race	4.4	38.9	17.3	47.0	
Asian	7.4	2.1	1.7	0.0	
Indigenous	0.0	0.0	0.0	2.0	
**Montdly income bracket**					< 0.003
Up to BRL 300	1.0	34.8	20.0	45.2	
BRL 300 - BRL 1.000	14.4	28.7	52.0	45.3	
BRL 1.000 - BRL 3.000	44.3	31.5	12.2	1.5	
BRL 3.000 - BRL 5.000	34.3	4.0	5,8	7.0	
tdey didn’t respond	6,0	1,0	10,0	1,0	
*Bolsa* *Família*					< 0.023
Yes	4.6	24.7	38.2	50.5	
**Family consumption level**					< 0.004
AB	81.4	52.4	27.3	6.8	
CD	18.6	47.6	72.7	93.2	

a) Chi-square test;

Most mothers had between 13 and 15 years of schooling (32.57%), followed by 16 years or more (31.42%). Years of study varied according to social stratum: mothers belonging to stratum D had mostly studied for less than 8 years (36.2%). Mothers belonging to stratum A had studied for the longest time, totaling 82.3% of those who studied for 16 years or more. In relation to maternal race/skin color, in the case of strata A, B and C, White race/skin color predominated, accounting for 85.8%, 57.9% and 70.0% of the total, respectively, while mixed race (47.0%) was predominant in stratum D (Table 2).

The proportion of children who received the *Bolsa* Família benefit varied between strata, being 4.6% in stratum A, 24.7% in stratum B, 38.2% in stratum C and 50.5% in stratum D. Regarding monthly family income, in stratum A, 44.3% had income between BRL 1,001 and BRL 3,000, in stratum B, 34.8% had income of up to BRL 300, in stratum C, 52.0% had income between BRL 301 and BRL 1,000, and in stratum D, 45.2% had income between BRL 301 and BRL 1,000. In relation to level of family consumption, 81.4% of families in stratum A had a level of consumption classified as AB (higher), while 93.2% of families in stratum D had a level of consumption classified as CD (lower) (Table 2). 

Vaccination coverage varied according to the social stratum of the families assessed. Children belonging to social stratum D had the highest vaccination coverage, reaching the PNI^4^ vaccine target: BCG (96.0%), Rotavirus (93.5%), 5-in-1 (97.0%), pneumococcal conjugate 10-valent (96.6%), meningococcal C (96.4%) and OPV (95.2%). The group of children belonging to stratum A only achieved the target^4^ recommended for BCG (93.6%) (Table 3).

**Table 3:** Coverage of vaccines recommended by 24 months old, by social strata, Londrina, Paraná, Brazil, 2021-2022 (n = 456)

**Vaccines scheduled** ^a^	**Social strata (95%CI)** ^b^			
	**A**	**B**	**C**	**D**
Bacillus Calmette-Guérin	93.6 (83.1;97.7)	96.8 (87.2;99.3)	93.2 (85.2;97.0)	96.0 (91.3;98.0)
Hepatitis B	89.2 (77.8;95.1)	81.2 (58.6;92.9)	90.5 (80.4;95.6)	83.9 (54.9;91.8)
Rotavirus	86.6 (77.9;95.5)	85.7 (61.0;95.8)	86.9 (72.4;94.4)	93.5 (86.0;97.2)
5-in-1	88.1 (76.7;94.3)	84.8 (60.9;95.2)	89.4 (77.7;95.4)	97.0 (91.4;99.1)
Inactivated poliovirus	51.3 (36.5;66.0)	81.0 (57.5;93.1)	85.4 (72.1;92.0)	94.5 (83.9;98.3)
Pneumococcal	88.0 (77.2;94.0)	82.7 (59.6;93.9)	83.7 (71.0;91.6)	96.6 (89.7;93.9)
Meningococcal C	76.7 (67.2;84.1)	81.0 (55.3;93.7)	85.3 (71.2;93.1)	96.4 (9.06;98.6)
First dose of oral poliovirus vaccine	53.7 (40.1;65.9)	94.6 (83.2;98.4)	89.8 (80.1;95.1)	95.2 (88.0;94.1)
Hepatitis A	93.0 (83.8;97.1)	98.4 (94.4;99.6)	91.3 (82.3;95.0)	84.3 (54.6;96.0)
Diphtderia + tetanus + pertussis	88.3 (77.2;94.3)	98.8 (95.0;99.7)	91.0 (82.0;95.6)	83.8 (55.1;95.6)
Measles + mumps + rubella	84.2 (74.6;90.1)	95.3 (80.6;99.0)	80.8 (56.7;93.1)	80.8 (78.5;93.2)

a) Vaccine against yellow fever was not considered in the analysis of coverage; b) Z test.

**Figure 1:**
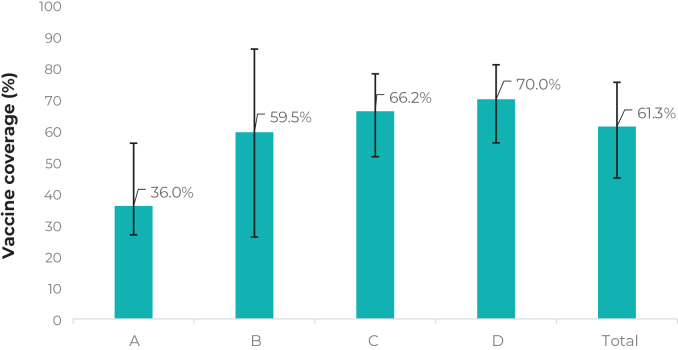
Vaccine coverage (full schedule, except yellow fever) scheduled by 24 months of life, by social strata, Londrina, Paraná, Brazil, 2021-2022 (n = 456)

Overall vaccination coverage was 61.3% (95%CI 44.9;75.4). The stratum with the lowest coverage was A with 36.0% (95%CI 26.8;57.8 ), followed by B with 59.5% (95%CI 26.1;86), C with 66.2% (95%CI 51.7;78.1) and D with 70.0 % (95%CI 56.1;81) (Figure 1).

**Figure 2:**
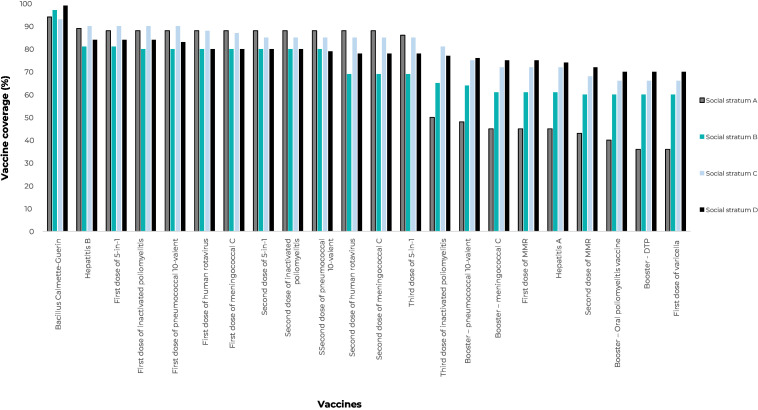
Evolution of vaccination coverage in children up to 24 months old, by social strata, Londrina, Paraná, Brazil, 2021-2022 (n = 456)

Vaccination coverage fulfilment demonstrated a reduction in doses administered over time in all social strata. In stratum A, vaccination coverage started at 93.6% for BCG and ended the child’s second year of life at 36.0% for varicella vaccination. In stratum D, the drops were less pronounced, starting with 99.0% for BCG and ending with 70.0% for varicella vaccination (Figure 2).

## DISCUSSION

This study identified that the vaccination schedule for children under 24 months of age did not reach the goals recommended by the PNI,^4^ except for the BCG vaccine. When analyzing vaccination coverage according to social stratum, it can be seen that the poorest coverage was found in the strata with the highest social status, while the best coverage was found in the least wealthy group, in line with the global trend,^14^ and exemplified by vaccination coverage in Latin American and Caribbean countries.^14,15^ In Europe, average vaccination coverage has also decreased and presents a high degree of heterogeneity between the different countries.^15^

The results of this study confirm previous findings indicating that census tracts with better social indicators have lower full vaccination coverage.^16^ Studies also reveal greater vaccination hesitancy and refusal among higher social strata, influenced by anti-vaccine movements that are harmful to vaccination coverage rates.^16,17^ Furthermore, families with better socioeconomic conditions may underestimate the importance of vaccines for child health, associating their circumstances with lower risk of infectious diseases.^1^

Another important fact about the relationship between vaccination coverage and social strata is that some studies have already demonstrated that in the case of a predominantly poor population, coverage is better among individuals who are part of the highest poverty group.^18^ This finding was seen in the population assessment of 45 less developed countries.^19,20^

Even in Brazilian regions where vaccination coverage reaches the targets recommended by the PNI,^6^ coverage is better among poorer populations. In 2007, in Curitiba, the capital of the state of Paraná, the full general vaccination schedule at 18 months reached 97.7%, but when analyzing by social stratum, the stratum with the lowest coverage was stratum A with 92.4%, followed by stratum B with 97.8%, C with 98.4% and D with 98.5%. Stratum E had the highest coverage (99.5%), and was the least wealthy stratum.^16^ This demonstrates the impact of health policies that follow the principles of the Brazilian National Health System (*Sistema* Único *de Saúde* - SUS), highlighting the importance of offering health services and immunization with equity.

It is noteworthy that the drops in vaccination coverage are more noticeable for vaccines that should be administered in the 4^th^ and 6^th^ months of life, a period in which children are more exposed to infectious diseases, such as mumps, rubella and measles, demonstrating the phenomenon of vaccination schedule dropout.^17^

Studies carried out in 2022 addressed this dropout throughout the child vaccination schedule. In relation to the period covering 2020 and 2021, the studies demonstrated that the abandonment rate was 42.26% in Macapá, 31.57% in Boa Vista, 29.19% in Porto Velho, 27.10% in Palmas, 26.95% in Manaus, 24.77% in Belém and 10.15% in Rio Branco.^21^ Considering dropout by type of vaccine, reduction rates of 0.9% were found for BCG, 1.3% for poliomyelitis and 2.7% for MMR. The Brazilian states that stand out in relation to vaccination schedule dropout were Pará, Maranhão and Bahia.^17,22^

Some Brazilian state capitals have a worrying impact on child vaccination schedule dropout, compromising the achievement of adequate vaccination coverage.^4^ In 2019 and 2020, there was 50% dropout in Manaus and Macapá, while Brasília, Teresina and Curitiba identified 20% and 10%.^7^ Regarding vaccines themselves, vaccination coverage was: 5-in-1 44.3%; IPV 51.7%; pneumococcal 54.6%, meningococcal C 58.2%; MMR first dose 58.2%, DTP 6.1% and OPV 36%.^18^

In Brazil, studies have indicated that between 2012 and 2021 vaccination coverage fell by 27.22% for the poliomyelitis vaccine, 26.01% for the first dose of the MMR vaccine, and 55.8% for the second MMR dose. Brazil has not achieved the target of 95.0% for the yellow fever vaccine since 2010. The 5-in-1 and MMR vaccines have also failed to reach this goal since 2013. In 2019 and 2020, the BCG vaccine also failed to reach its target, highlighting a sharp reduction in coverage since 2016.^8^

Results of the last household vaccination survey carried out in Brazil, in 2007, showed that the full vaccination schedule did not meet the recommended levels needed to increase protection against the risk of infectious diseases in children under 24 months old.^4,6^

In the present study, vaccination schedule dropout was greater in the population belonging to the highest social stratum, demonstrating that this population must be considered potentially vulnerable, also resulting in an increase in risk for populations belonging to lower social strata, as a result of failure to achieve better control of vaccine-preventable diseases. This phenomenon increases the number of susceptible children, in addition to variations depending on availability of resources and socioeconomic status.^24^ Considering the population characteristics of the city of Londrina, it can be inferred that the results indicate that parents and guardians in the highest social stratum end up not placing value on the vaccination status of children under 24 months old. A possible justification may be their need to undertake activities away from home, such as work and studies. Another possibility may relate to the organization of the health system which seeks to benefit people with greater social vulnerability and considers that population groups with greater purchasing power have developed greater social and health awareness.

Another weakness identified in the literature is the lack of health professionals with adequate knowledge of when vaccines should be administered and when they can be administered simultaneously, which impacts professional safety, family confidence and results in the loss of opportunities to keep vaccination up to date.^17^ Another point to be highlighted is that between 2015 and 2017 there was also shortage and lack of vaccine in health service facilities needed in order to provide vaccination,^25^ which occurred due to limited vaccine production capacity and the increase in global demand affecting BCG, MMR, 5-in-1 and rotavirus vaccines.^8^

Current challenges to improving the achievement of the goals recommended by the PNI are related to people’s lack of awareness regarding the importance of infectious diseases, growing demands on the health service for the care of acute diseases, weakness in communication with the population about the safety and benefits of vaccines, significant increase in information that causes distrust about vaccines, incipient provision of extramural vaccination, problems with quality of records and difficulties with information systems.^5^

In order to address the complexity of the challenges in maintaining adequate vaccination coverage, the use of strategic planning, considering the local context, can contribute to improving the results found in this study, associated with the consolidation of organizational technologies for health services and expanded humanization capabilities to attend to the needs of the local population and improve timely vaccination, thus avoiding missed opportunities.^23^ Strengthening communication to provide safe, reliable and up-to-date information about the benefits of vaccination is identified as one of the successful strategies.^15^

 Programs aimed at children and which use home visits as a strategy have been shown to contribute to increasing the vaccination rate.^18^ It is also noteworthy that training health professionals to develop strategies that seek to improve vaccination coverage has proven to be an important pathway to be considered. A nursing professional who is qualified not only in administering vaccinations, but also in building bonds with families and having effective communication skills to advocate for vaccination, has proven to be a strategy resulting in better vaccination coverage.^8^

A limitation of this study lies in difficulties in reading vaccination cards, since there is no standardization of the models used, as well as the presence of errors and records that made reading difficult, which could generate interpretation bias. To avoid bias, data were collected by health professionals duly trained in immunization. Another limitation was the use of outhated 2010 census data.

In conclusion, the results provided greater clarity as to the situation of vaccination, as well as demonstrating that achieving the goals established by the PNI is a major challenge. Social stratum A, considered the wealthiest, was the stratum most vulnerable to vaccine-preventable diseases, as well as having most effect on fulfilment of the vaccination schedule by 24 months old. These findings reinforce the ongoing need to qualify the vaccination program and enable greater access to vaccines for children, as well as being robust evidence to support local health service managers and professionals in strengthening communication strategies with the population about the risks of vaccine-preventable diseases and enable greater PNI efficiency.

Use of the results of this study as triggers for ongoing health education processes is recommended, with the aim of stimulating dialogue based on discussions and reflections on vaccination practice, seeking to identify the contextualized causes of low coverage, as well as enabling the collective building of local strategies to achieve the targets established by the PNI, thus encouraging possibilities of establishing alternative and innovative strategies based on the perceptions of health professionals and the community.
