# Supplementary material for: Vaccination coverage survey by social stratum in children up to 24 months of age in Londrina, Paraná, Brazil, between 2021 and 2022
Source: Epidemiol Serv Saude. 2024 Oct 21;33(spe2):e20231393. doi: 10.1590/S2237-96222024v33e20231393.especial2.en (PMC11493398; doi:10.1590/S2237-96222024v33e20231393.especial2.en)
Supplement: Translation

# Translation: Inquérito de cobertura vacinal segundo estrato social em crianças até 24 meses de vida, em Londrina, Paraná, entre 2021 e 2022

**DOI:** 10.1590/S2237-96222024v33e20231393.especial2.pt

**Authors:** Edmilson de Oliveira, José Cássio de Moraes, Ana Paula França, Grupo ICV 2020, Adriana Ilha da Silva, Alberto Novaes Ramos, Ana Paula França, Andrea de Nazaré Marvão Oliveira, Antonio Fernando Boing, Carla Magda Allan Santos Domingues, Consuelo Silva de Oliveira, Ethel Leonor Noia Maciel, Ione Aquemi Guibu, Isabelle Ribeiro Barbosa Mirabal, Jaqueline Caracas Barbosa, Jaqueline Costa Lima, José Cássio de Moraes, Karin Regina Luhm, Karlla Antonieta Amorim Caetano, Luisa Helena de Oliveira Lima, Maria Bernadete de Cerqueira Antunes, Maria da Gloria Teixeira, Maria Denise de Castro Teixeira, Maria Fernanda de Sousa Oliveira Borges, Rejane Christine de Sousa Queiroz, Ricardo Queiroz Gurgel, Rita Barradas Barata, Roberta Nogueira Calandrini de Azevedo, Sandra Maria do Valle Leone de Oliveira, Sheila Araújo Teles, Silvana Granado Nogueira da Gama, Sotero Serrate Mengue, Taynãna César Simões, Valdir Nascimento, Wildo Navegantes de Araújo

**Affiliations:** 1 Santa Casa de São Paulo, Faculdade de Ciências Médicas, São Paulo, SP, Brasil; Universidade Federal do Espírito Santo, Vitória, ES, Brasil; Universidade Federal do Ceará, Departamento de Saúde Comunitária, Fortaleza, CE, Brasil; Faculdade Ciências Médicas Santa Casa de São Paulo, São Paulo, SP, Brasil; Secretaria de Estado da Saúde do Amapá, Macapá, AP, Brasil; Universidade Federal de Santa Catarina, SC, Brasil; Organização Pan-Americana da Saúde, Brasília, DF, Brasil; Instituto Evandro Chagas, Belém, PA, Brasil; Faculdade de Ciências Médicas Santa Casa de São Paulo, Departamento de Saúde Coletiva, São Paulo, SP, Brasil; Universidade Federal do Rio Grande do Norte, Natal, RN, Brasil; Universidade Federal do Ceará, Departamento de Saúde Comunitária, Fortaleza, CE, Brasil; Universidade Federal de Mato Grosso, Cuiabá, MT, Brasil; Universidade Federal do Paraná, Curitiba, PR, Brasil; Universidade Federal de Goiás, Goiânia, GO, Brasil; Universidade Federal do Piauí, Teresina, PI, Brasil; Universidade de Pernambuco, Faculdade de Ciências Médicas, Pernambuco, PE, Brasil; Instituto de Saúde Coletiva, Universidade Federal da Bahia, Salvador, BA, Brasil; Secretaria de Estado da Saúde de Alagoas, Maceió, AL, Brasil; Universidade Federal do Acre, Rio Branco, AC, Brasil; Universidade Federal do Maranhão, Departamento de Saúde Pública, São Luís, MA, Brasil; Universidade Federal de Sergipe, Aracaju, SE, Brasil; Secretaria Municipal de Saúde, Boa Vista, RR, Brasil; Fundação Oswaldo Cruz, Mato Grosso do Sul, Campo Grande, MS, Brasil; Fundação Oswaldo Cruz, Escola Nacional de Saúde Pública Sergio Arouca, Rio de Janeiro, RJ, Brasil; Universidade Federal do Rio Grande do Sul, Porto Alegre, RS, Brasil; Fundação Oswaldo Cruz, Instituto de Pesquisa René Rachou, Belo Horizonte, MG, Brasil; Secretaria de Desenvolvimento Ambiental de Rondônia, Porto Velho, RO, Brasil; Universidade de Brasília, Brasília, DF, Brasil

**Keywords:** Cobertura Vacinal, Esquema de Imunização, Inquéritos Epidemiológicos, Avaliação de Programa

## Abstract

**Objetivo:**

Analisar a cobertura vacinal segundo estrato social, em crianças até 24 meses de vida, residentes no município de Londrina (PR).

**Métodos:**

Inquérito de base populacional, realizado entre em 2021 e 2022, em que se avaliou a cobertura vacinal e os aspectos sociodemográficos das mães e das famílias, através do teste qui-quadrado de Pearson.

**Resultados:**

Em uma amostra de 456 crianças, a cobertura vacinal completa variou de acordo com o estrato social, sendo no estrato A de 36,0% (IC_95_% 26,8;57,8); no estrato B, de 59,5% (IC_95_% 26,1;86); no estrato C, de 66,2% (IC_95_% 51,7;78,1); e no estrato D, de 70,0% (IC_95_% 56,1;81,0).

**Conclusão:**

A análise da cobertura vacinal indicou o estrato social A como o de maior risco para doenças prevenidas por vacinas.

## Contribuições do estudo

**Principais resultados:**

Os resultados do estudo evidenciaram cobertura vacinal completa baixa em crianças até 24 meses de vida em Londrina, sendo maior no estrato social menos favorecido financeiramente, comparado ao mais favorecido.

**Implicações para os serviços:**

Os resultados encontrados podem subsidiar a qualificação do programa de imunização e possibilitar, a partir de planejamento e educação permanente em saúde, a definição de estratégias singularizadas para melhorar a cobertura vacinal.

**Perspectivas:**

Como perspectivas futuras, sinaliza-se a importância da realização de investigações a respeito dos desafios inerentes à vacinação, bem como de pesquisas qualitativas e quantitativas abordando profissionais de saúde para melhor compreensão dos dados.

## INTRODUÇÃO

O conhecimento preciso da cobertura vacinal em crianças é um dos elementos importantes para a avaliação dos programas de vigilância epidemiológica, na medida em que permite acompanhar o aumento do número de indivíduos suscetíveis na população, bem como avaliar a efetividade da imunidade adquirida como barreira para a transmissão de doenças prevenidas por vacinas.^1^

As vacinas são importantes investimentos em saúde pública, por prevenirem doenças infecciosas, reduzirem a gravidade das doenças e apoiarem na redução da mortalidade infantil, apresentando alta eficácia, segurança e custo-benefício.^2^ Nos últimos 20 anos, houve grande comprometimento dos gestores em saúde visando ao avanço global dos programas de vacinação, que obtiveram sucesso em países pobres, com mais de 300 milhões de crianças imunizadas e cerca de 4 milhões de crianças salvas.^3^

No Brasil, o Programa Nacional de Imunizações (PNI) é a instância responsável por promover o controle de doenças transmissíveis por meio da vacinação de rotina, campanhas de vacinação periódicas e vigilância epidemiológica de doenças transmissíveis.^4^ Ainda é um desafio sustentar as coberturas vacinais em níveis recomendados para garantir a prevenção da saúde das crianças,^5^ com metas recomendadas de 90% para as vacinas Bacilo Calmette-Guérin (BCG) e contra rotavírus humano; e de 95% para as vacinas contra a poliomielite, contra a difteria, tétano e coqueluche (DTP), contra o sarampo, caxumba e rubéola (tríplice viral), contra a hepatite B, contra a febre amarela, vacina pentavalente, vacina meningocócica C conjugada, vacina pneumocócica 10-valente, vacina contra a varicela, vacina contra a hepatite A e vacina tetravalente.^4^

O último inquérito nacional domiciliar de cobertura vacinal, realizado em 2007 nas capitais brasileiras e no Distrito Federal, mostrou os seguintes percentuais de cobertura: 97% para BCG, 96% para poliomielite, 94% para DTP, 91% para hepatite B e 91% para tríplice viral, indicando que apenas as vacinas BCG e poliomielite alcançaram os parâmetros recomendados.^4,6^ Quanto ao esquema de vacinação completo para doses válidas, aquelas aplicadas no período recomendado pelo PNI, o estudo identificou uma cobertura geral de 68,0%.^7^

Além de investigar a cobertura vacinal, estudos têm buscado compreender como os determinantes sociais interferem na vacinação em crianças. No Brasil, entre o período de 2010 e 2020, as menores coberturas vacinais e maiores tendências à queda se relacionaram aos menores índices de desenvolvimento humano, concentrados nas regiões Norte e Nordeste.^8^

Para enfrentar essas disparidades, estudos do tipo inquérito de cobertura vacinal são ferramentas essenciais, já que possibilitam demonstrar a situação vacinal de forma fidedigna; identificar o número de suscetíveis para doenças infecciosas; auxiliar a compreender o acesso e a adesão ao PNI; apoiar na análise e confiabilidade dos dados administrativos; bem como investigar as condições de vida que interferem na cobertura vacinal, buscando identificar as desigualdades sociais em saúde.^7^

Considerando a importância da manutenção de boas coberturas vacinais para a promoção da saúde das crianças até 24 meses de vida e a necessidade do conhecimento da situação vacinal segundo o *status* social para compreensão dos desafios atuais a serem enfrentados, o presente estudo teve por objetivo analisar a cobertura vacinal de acordo com o estrato social em crianças até 24 meses de vida, residentes no município de Londrina (PR).

## MÉTODOS

Trata-se de um estudo do tipo inquérito de base domiciliar que identificou a cobertura vacinal em crianças até 24 meses de vida, realizado nos meses de outubro de 2021 a janeiro de 2022. Esta pesquisa faz parte de uma investigação ampliada denominada “Inquérito de Cobertura Vacinal em municípios com mais de 200.000 habitantes”.

O estudo foi realizado no município de Londrina, localizado na região norte do Paraná, cuja população residente é de 575.377 habitantes e a densidade demográfica de 306,52 habitantes/km.^9^ A organização da Atenção Primária em Saúde conta com 54 unidades básicas de saúde e 74 salas de vacina.^10^

Como critério de inclusão, definiu-se crianças residentes na área urbana do município de Londrina, nascidas vivas em 2017 e 2018. A base populacional foi obtida pelo Sistema de Informações sobre Nascidos Vivos (Sinasc) e o período de seguimento foi retrospectivo. Os recém-nascidos foram georreferenciados nos setores censitários de residência,^11^ obtidos através do Censo Demográfico 2010.^9^

O tamanho da amostra levou em conta o efeito do desenho pela utilização de conglomerados de setores censitários, estabelecido em 1,4, com base em estudos anteriores; população hipotética de 14 mil nascidos vivos (NV); prevalência estimada de cobertura vacinal de 70%, erro de estimativa de 5% e 1,96 para intervalo de confiança de 95%.^12^ A amostra foi alocada em quatro estratos sociais dos setores censitários, formada por aproximadamente 113 crianças. Além de utilizar procedimentos de alocação desproporcional, foi necessário calcular e utilizar pesos amostrais para cada domicílio entrevistado, de modo a permitir a estimação sem viés dos parâmetros de interesse na população.^12^

Os dados usados na classificação dos setores censitários foram: renda média dos responsáveis pelo domicílio, proporção de responsáveis alfabetizados e proporção de responsáveis com renda maior ou igual a 20 salários mínimos; em seguida, foram divididos nos estratos sociais A (alto), B (médio-alto), C (médio-baixo) e D (baixo). Os setores foram agrupados por meio da análise de *cluster* em conglomerados.^12^

Para identificar o nível de consumo familiar, as famílias foram classificadas de acordo com os critérios da Associação Brasileira de Empresas de Pesquisa (ABEP),^13^ como: A, B, C e D, sendo A o nível de consumo mais alto e D o mais baixo. As características do domicílio consideradas foram: número de pessoas residentes no domicílio, número de cômodos servindo de dormitório, número de banheiros de uso exclusivo dos residentes, número de automóveis de passeio de uso exclusivo familiar, número de motocicletas para uso exclusivo, número de empregados mensalistas, número de geladeiras, *freezers*, máquinas de lavar roupa, de lavar louça, fornos de micro-ondas, secadoras de roupa, leitores de DVD e microcomputadores.^12^

A coleta dos dados foi feita por empresa especializada em realização de inquéritos domiciliares, seguindo os requisitos legais para a contratação, considerando que os pesquisadores de campo tivessem formação na área da saúde, experiência em coleta de dados e conhecimento em imunização. As variáveis foram obtidas por aplicação de um questionário e as doses aplicadas de vacina foram obtidas por uma foto legível da(s) caderneta(s) de vacinação da criança. Essas datas foram lidas por profissionais de saúde que trabalham com vacina e transcritas para o questionário eletrônico. 

Em relação às características das crianças, as variáveis avaliadas foram: sexo (masculino e feminino) e raça/cor da pele autodeclarada (branca, preta, parda, amarela e indígena). As características das mães foram: faixa etária da mãe (< 20 anos, 20 a 34 anos e acima de 35 anos), escolaridade (até 8 anos, 9 a 12 anos, 13 a 15 anos e 16 anos ou mais), raça/cor da pele autodeclarada (branca, preta, parda, amarela e indígena) e Bolsa Família (sim). Para renda familiar mensal, considerou-se: até R$ 300; R$ 301 até R$ 1.000; R$ 1.001 a R$ 3.000; R$ 3.001 a R$ 5.000. Ao se avaliarem os níveis de consumo separadamente, identificou-se pequena proporção no nível A, optando-se por agrupar os níveis A e B; e C e D.

As vacinas incluídas no estudo foram: BCG, hepatite B, rotavírus, pentavalente, vacina poliomielite Inativada (VIP), pneumocócica, meningocócica C, vacina poliomielite oral (VOP), hepatite A, DTP e tríplice viral (contra sarampo, caxumba e rubéola). Considerou-se como esquema completo aos 24 meses de vida a recomendação do PNI, que define como completo o esquema vacinal quando pelo menos 90% das crianças receberam as vacinas BCG (uma dose) e rotavírus humano (duas doses), e 95% receberam as vacinas contra poliomielite (três doses e um reforço), DTP (três doses e um reforço), tríplice viral (duas doses), hepatite B (uma dose), febre amarela (uma dose), pentavalente (três doses), meningocócica C conjugada (três doses), pneumocócica 10-valente (três doses), varicela (uma dose), hepatite A (uma dose) e tetraviral (uma dose).^4^

O cálculo da cobertura vacinal foi realizado considerando-se como numerador o número de vacinas aplicadas e como denominador o quantitativo de crianças até 24 meses de vida no município de Londrina. Também foi calculada a proporção de crianças vacinadas em cada estrato social, identificando-se a cobertura em cada estrato analisado.

Todas as análises foram realizadas no programa Stata®, versão 17, utilizando-se o módulo survey analysis, considerando-se os pesos amostrais e o delineamento do estudo. Para identificar diferenças estatísticas, as coberturas vacinais foram apresentadas com os respectivos IC_95_% e as diferenças nas proporções das variáveis entre os grupos de crianças foram avaliadas utilizando-se o teste qui-quadrado de Pearson com significância de 5% (p < 0,05).

Os participantes do estudo assinaram o Termo de Consentimento Livre e Esclarecido, e a confidencialidade dos dados foi mantida pelos pesquisadores envolvidos. O estudo foi aprovado pelos Comitês de Ética em Pesquisa com Seres Humanos do Instituto de Saúde Coletiva da Universidade Federal da Bahia, sob parecer nº 3.366.818, em 4 de junho de 2019, com Certificado de Apresentação de Apreciação Ética (CAAE) 4306919.5.0000.5030, e da Irmandade da Santa Casa de São Paulo, sob parecer nº 4.380.019, em 4 de novembro de 2020, com CAAE 39412020.0.0000.5479. 

## RESULTADOS

Participaram do estudo 456 crianças, obtidas a partir de uma população de nascidos vivos em 2017 e 2018 no município de Londrina, de 14.091 crianças. O estudo incluiu 3,1% dessa população, não havendo perdas. Do total de crianças da amostra, 56,0% eram do sexo masculino e 44,0% do sexo feminino. Quanto à raça/cor da pele, 65,0% foram declaradas como brancas; 30,0%, pardas; 3,0%, pretas; 1,0%, indígenas; e 1,0%, amarelas (Tabela 1).

**Tabela 1:** Características sociodemográficas das crianças até 24 meses de vida, segundo estrato social, Londrina, Paraná, 2021-2022 (n = 456)

**Características**	**Estrato social (%)**					
	A (115)	B (114)	C (113)	D (114)	Total (456)	p-valor ^a^
**Sexo**						< 0,205
Masculino	57,7	53,4	62,3	48,0	56,0	
Feminino	42,3	46,6	37,7	52,0	44,0	
**Raça/cor da pele**						< 0,388
Branca	89,0	61,0	77,0	43,5	65,0	
Preta	0,0	0,0	8,0	4,0	3,0	
Parda	6,0	38,0	15,0	52,5	30,0	
Amarela	5,0	0,0	0,0	0,0	1,0	
Indígena	0,0	1,0	0,0	0,0	1,0	

a) Teste qui-quadrado.

A faixa etária das mães mais prevalente foi a de 20 a 34 anos, representando 29,0% das mães do estrato A, 44,4% do estrato B, 71,9% do estrato C e 76,1% do estrato D. Mães com menos de 20 anos foram encontradas em maior proporção no estrato B (21,8%). Já o grupo de mães com 35 anos ou mais foi mais prevalente no estrato A, representando 70,0% do total (Tabela 2). 

**Tabela 2:** Características maternas e das familias das crianças até 24 meses de vida, segundo estrato social, Londrina, Paraná, 2021-2022 (n = 456)

**Características**	**Estrato social (%)**				
	A (n = 115)	B (n = 114)	C (n = 113)	D (n = 114)	p-valor ^a^
**Faixa etária materna (em anos)**					< 0,001
Abaixo de 20	1,0	21,8	2,2	2,4	
20-34	29,0	44,4	71,9	76,1	
35 ou mais	70,0	33,8	25,9	21,5	
**Escolaridade (em anos de estudo)**					< 0,003
Até 8	1,0	33,1	15,6	36,2	
9 a 12	2,4	2,0	16,5	22,4	
13 a 15	13,6	25,1	49,0	30,3	
16 ou mais	82,3	39,3	17,9	9,3	
**Raça/cor da pele**					< 0,032
Branca	85,8	57,9	70,0	39,0	
Preta	2,4	1,1	11,0	12,0	
Parda	4,4	38,9	17,3	47,0	
Amarela	7,4	2,1	1,7	0,0	
Indígena	0,0	0,0	0,0	2,0	
**Faixa de renda mensal**					< 0,003
Até R$ 300	1,0	34,8	20,0	45,2	
R$ 300 a R$ 1.000	14,4	28,7	52,0	45,3	
R$ 1.000 a R$ 3.000	44,3	31,5	12,2	1,5	
R$ 3.000 a R$ 5.000	34,3	4,0	5,8	7,0	
Sem resposta	6,0	1,0	10,0	1,0	
**Bolsa Família**					< 0,023
Sim	4,6	24,7	38,2	50,5	
**Nível de consumo familiar**					< 0,004
AB	81,4	52,4	27,3	6,8	
CD	18,6	47,6	72,7	93,2	

a) Teste qui-quadrado.

A maioria das mães estudou entre 13 e 15 anos (32,57%), vindo em seguida a faixa de 16 anos ou mais (31,42%). Os anos de estudo variaram de acordo com o estrato social: mães pertencentes ao estrato D estudaram em sua maioria menos de 8 anos (36,2%). As mães pertencentes ao estrato A foram as que tiveram maior tempo de estudo, totalizando 82,3% das que estudaram 16 anos ou mais. Em relação à raça/cor da pele da mãe, para os estratos A, B e C, predominaram a raça/cor da pele branca, com respectivamente 85,8%, 57,9% e 70,0% do total, enquanto para o estrato D sobressaiu a parda, com 47,0% (Tabela 2).

A proporção de crianças que recebiam o benefício do programa Bolsa Família variou entre os estratos, sendo 4,6% no estrato A, 24,7% no estrato B, 38,2% no estrato C e 50,5% no estrato D. Em relação à renda mensal familiar, no estrato A, 44,3% tinham renda entre R$ 1.001 e R$ 3.000, no estrato B, 34,8% tinham renda até R$ 300, no estrato C, 52,0% tinham renda entre R$ 301 e R$ 1.000, e no estrato D, 45,2% tinham renda entre R$ 301 e R$ 1.000. Em relação ao nível de consumo familiar, 81,4% das famílias do estrato A tiveram um nível de consumo classificado como AB (maior), enquanto 93,2% das famílias do estrato D tiveram um nível de consumo classificado como CD (menor). (Tabela 2). 

A cobertura vacinal variou de acordo com o estrato social das famílias avaliadas. As crianças pertencentes ao estrato social D apresentaram as maiores coberturas vacinais, alcançando a meta do PNI^4^ para as vacinas: BCG (96,0%), rotavírus (93,5%), pentavalente (97,0%), vacina pneumocócica conjugada 10-valente (96,6%), meningo C (96,4%) e VOP (95,2%). Já o grupo de crianças pertencentes ao estrato A somente alcançou a meta^4^ recomendada para a vacina BCG (93,6%) (Tabela 3).

**Tabela 3:** Cobertura das vacinas recomendadas até 24 meses de vida, segundo estrato social, Londrina, Paraná, 2021-2022 (n = 456)

Vacinas previstas^a^	Estrato social (IC_95_%)^b^			
	A	B	C	D
Bacilo Calmette-Guerin	93,6 (83,1;97,7)	96,8 (87,2;99,3)	93,2 (85,2;97,0)	96,0 (91,3;98,0)
Hepatite B	89,2 (77,8;95,1)	81,2 (58,6;92,9)	90,5 (80,4;95,6)	83,9 (54,9;91,8)
Rotavírus	86,6 (77,9;95,5)	85,7 (61,0;95,8)	86,9 (72,4;94,4)	93,5 (86,0;97,2)
Pentavalente	88,1 (76,7;94,3)	84,8 (60,9;95,2)	89,4 (77,7;95,4)	97,0 (91,4;99,1)
Poliomielite inativada	51,3 (36,5;66,0)	81,0 (57,5;93,1)	85,4 (72,1;92,0)	94,5 (83,9;98,3)
Pneumocócica	88,0 (77,2;94,0)	82,7 (59,6;93,9)	83,7 (71,0;91,6)	96,6 (89,7;93,9)
Meningocócica C	76,7 (67,2;84,1)	81,0 (55,3;93,7)	85,3 (71,2;93,1)	96,4 (9,06;98,6)
Primeira dose da vacina oral poliomielite	53,7 (40,1;65,9)	94,6 (83,2;98,4)	89,8 (80,1;95,1)	95,2 (88,0;94,1)
Hepatite A	93,0 (83,8;97,1)	98,4 (94,4;99,6)	91,3 (82,3;95,0)	84,3 (54,6;96,0)
Difteria + tétano + coqueluche	88,3 (77,2;94,3)	98,8 (95,0;99,7)	91,0 (82,0;95,6)	83,8 (55,1;95,6)
Tríplice viral	84,2 (74,6;90,1)	95,3 (80,6;99,0)	80,8 (56,7;93,1)	80,8 (78,5;93,2)

a) Não foi considerada, na análise de cobertura, a vacina contra febre amarela; b) Teste Z.

A cobertura vacinal geral foi de 61,3% (IC_95_% 44,9;75,4), sendo o estrato de menor cobertura o A, com 36,0% (IC_95_% 26,8;57,8), seguido pelo B (59,5%; IC_95_% 26,1;86), C (66,2% IC_95_% 51,7;78,1) e D (70,0%; IC_95_% 56,1;81) (Figura 1).

**Figura 1:** Cobertura das vacinas (esquema completo, exceto febre amarela) previstas no calendário até 24 meses de vida, segundo estrato social, Londrina, Paraná, 2021-2022 (n = 456)

O seguimento da cobertura vacinal demonstrou uma redução nas doses aplicadas ao longo do tempo em todos os estratos sociais. No estrato A, a cobertura vacinal se iniciou em 93,6% para a vacina BCG e finalizou o segundo ano de vida da criança com 36,0% para a vacina contra varicela. Já no estrato D, as quedas foram menos acentuadas, iniciando-se com 99,0% para a vacina BCG e finalizando com 70,0% para a vacina contra varicela (Figura 2).

**Figura 2:** Evolução da cobertura vacinal em crianças até 24 meses de vida, segundo estrato social, Londrina, Paraná, 2021-2022 (n = 456)

## DISCUSSÃO

Este estudo identificou que o esquema de vacinação para menores de 24 meses de vida não atingiu as metas preconizadas pelo PNI,^4^ exceto para a vacina BCG. Ao se analisar a cobertura vacinal segundo estrato social, nota-se que as piores coberturas foram observadas nos estratos de melhor *status* social e as melhores coberturas no grupo de menor condição financeira, dialogando com a tendência global,^14^ e exemplificada pelas coberturas vacinais nos países da América Latina e do Caribe.^14,15^ Na Europa, a média das coberturas vacinais também diminuiu e apresenta alto grau de heterogeneidade entre os diferentes países.^15^

Os resultados deste estudo confirmam achados anteriores, indicando que os setores censitários com melhores indicadores sociais têm menor cobertura vacinal completa.^16^ Estudos também revelam maior hesitação vacinal e recusa entre estratos sociais mais altos, influenciados por movimentos antivacina que prejudicam as taxas de cobertura vacinal.^16,17^ Além disso, famílias com melhores condições socioeconômicas podem subestimar a importância das vacinas para a saúde infantil, associando suas circunstâncias a um menor risco de doenças infecciosas.^1^

Outro fato importante sobre a relação entre a cobertura vacinal e o estrato social é que alguns estudos já demonstraram que, para uma população predominantemente pobre, a cobertura é melhor nos indivíduos que fazem parte do grupo de maior pobreza,^18^ achado este observado na avaliação da população de 45 países menos desenvolvidos.^19,20^

Mesmo em regiões do país onde a cobertura vacinal atinge as metas preconizadas pelo PNI,^6^ a cobertura é melhor em populações mais pobres. Em 2007, na capital do Paraná, Curitiba, o esquema de vacinação geral completo aos 18 meses foi de 97,7%. Analisando por estrato social, o estrato com menor cobertura foi o A, com 92,4%, seguido pelo estrato B, com 97,8%, C, com 98,4% e o D, com 98,5%. O estrato com maior cobertura foi o estrato E com 99,5%, sendo esse o de menor riqueza.^16^ Isso demonstra o impacto de políticas de saúde que consideram os princípios do Sistema Único de Saúde (SUS), destacando a importância da oferta de serviço de saúde e de imunização com equidade.

Vale destacar que as quedas nas coberturas vacinais são mais perceptíveis para as vacinas que deveriam ser aplicadas no 4º e no 6º mês de vida, período em que as crianças estão mais expostas a doenças infecciosas, como caxumba, rubéola e sarampo, demonstrando o fenômeno do abandono do esquema vacinal.^17^

Estudos realizados em 2022 abordaram o abandono ao longo do esquema de vacinação das crianças. O período de 2020 a 2021 demonstrou que em Macapá a proporção de abandono foi de 42,26%; em Boa Vista, de 31,57%; em Porto Velho, de 29,19%; em Palmas, de 27,10%; em Manaus, de 26,95%; em Belém, de 24,77%; e em Rio Branco, de 10,15%.^21^ Considerando-se o abandono por tipo de vacina, para a vacina BCG, a redução chega a 0,9%; poliomielite, a 1,3%; e tríplice viral a 2,7%. Os estados do Brasil que se destacavam em relação ao abandono do esquema vacinal foram Pará, Maranhão e Bahia.^17,22^

Algumas capitais do Brasil apresentam impacto preocupante do abandono do esquema de vacinação em crianças, prejudicando o alcance das coberturas vacinais adequadas.^4^ Em 2019 e 2020, Manaus e Macapá obtiveram abandono de 50%, já Brasília, Teresina e Curitiba identificaram 20% e 10%.^7^ Em relação às vacinas, a cobertura vacinal foi: para a pentavalente, de 44,3%; VIP, de 51,7%; pneumocócica, de 54,6%; meningo C, de 58,2%; primeira dose da tríplice viral, de 58,2%; tríplice bacteriana, de 6,1%; e VOP, de 36%.^18^

No Brasil, estudos indicaram que, entre 2012 e 2021, houve redução na cobertura vacinal de 27,22%; para a vacina contra poliomielite, 26,01% para a primeira dose da tríplice viral, e 55,8% para a segunda dose. Desde 2010, o país não atinge a meta de 95,0% para a vacina contra a febre amarela; desde 2013, as vacinas pentavalente e tríplice viral também não alcançaram essa meta. Em 2019 e 2020, a vacina BCG também não alcançou a meta, destacando-se acentuada redução das coberturas desde 2016.^8^

Resultados do último inquérito domiciliar vacinal realizado no Brasil, em 2007, mostraram que o esquema vacinal completo não alcançou as recomendações para aumentar a proteção diante do risco de doenças infecciosas em crianças menores de 24 meses de vida.^4,6^

No presente estudo, o abandono do esquema vacinal foi maior na população do estrato social mais alto, demonstrando que essa população deve ser considerada potencialmente vulnerável, tendo como consequência o aumento do risco também para populações dos estratos sociais mais baixos, por não se estabelecer um melhor controle das doenças imunopreveníveis. Esse fenômeno aumenta o número de crianças suscetíveis, e existem variações de acordo com a disponibilidade de recursos e situação socioeconômica.^24^ Considerando-se a característica populacional do município de Londrina, pode-se inferir que os resultados indicam que esse grupo de responsáveis pelas crianças acabam não valorizando o *status* vacinal das crianças menores de 24 meses de vida. Uma possível justificativa pode estar na necessidade de desenvolver atividades fora do domicílio, como trabalho e estudo. Outra possibilidade pode estar em torno da organização do sistema de saúde, que busca beneficiar a população de maior vulnerabilidade social e considera que grupos populacionais de maior poder aquisitivo tenham desenvolvido maior consciência social e de saúde.

Outra fragilidade identificada na literatura é a falta de profissionais com conhecimento adequado da indicação das vacinas e a respeito de aplicação simultânea, o que impacta a segurança do profissional e a confiança dos familiares, e resulta na perda de oportunidade para atualizar o esquema vacinal.^17^ Destaca-se também o desabastecimento e a falta de vacina nas unidades para realizar a vacinação,^25^ que ocorreu devido à capacidade limitada na produção de vacinas e ao aumento da demanda mundial, afetando as vacinas BCG, tríplice viral, pentavalente e rotavírus, entre 2015 e 2017.^8^

Os desafios atuais para melhorar o alcance das metas preconizadas pelo PNI se relacionam à falta de consciência da população com relação à importância das doenças infecciosas, às demandas crescentes no serviço de saúde para o atendimento de doenças agudas, à fragilidade na comunicação com a população sobre a segurança e o benefício das vacinas, ao aumento significativo de informações que provocam desconfiança sobre as vacinas, à oferta de vacinação extramuro incipiente, aos problemas com qualidade de registros e às dificuldades com sistemas de informação.^5^

Para lidar com a complexidade dos desafios em manter coberturas vacinais adequadas, o uso do planejamento estratégico, considerando o contexto local, pode contribuir para melhorar os resultados encontrados neste estudo, associado à consolidação de tecnologias organizacionais para serviços de saúde e capacidades ampliadas de humanização, para acolher as necessidades da população local e melhorar a vacinação oportuna, evitando as perdas de oportunidade.^23^ O fortalecimento da comunicação para o fornecimento de informações seguras, confiáveis e atualizadas sobre os benefícios da vacinação é apontado como umas das estratégias de sucesso.^15^

 Programas voltados para crianças e que consideram como estratégia a visita domiciliar contribuíram com o aumento da taxa de vacinação.^18^ Ressalta-se ainda que a capacitação dos profissionais de saúde para desenvolver estratégias que busquem melhorar as coberturas vacinais vem se mostrando um caminho importante a ser considerado. O profissional de enfermagem qualificado não só para a execução da vacina, mas também para a construção de vínculo com a família e habilidades de comunicação efetiva em defesa da vacinação, mostrou-se uma estratégia que resultou em melhores coberturas vacinais.^8^

Como limitações deste estudo, podemos indicar dificuldades na leitura da caderneta de vacinação, uma vez que não existe padronização dos modelos utilizados, e presença de erros e registros que dificultaram a leitura, podendo gerar viés de interpretação; também utilizamos o censo desatualizado. Para evitar os vieses, os dados foram coletados por profissionais de saúde devidamente treinados em imunização.

Conclui-se que os resultados demonstraram a situação vacinal com maior clareza e que atingir as metas estabelecidas pelo PNI é um grande desafio. O estrato social A, considerado o mais rico, foi o que apresentou maior vulnerabilidade para doenças prevenidas por vacinas, assim como maior efeito de seguimento do calendário de vacinação até os 24 meses de vida. Esses achados reforçam a necessidade contínua de qualificar o programa de vacinação e possibilitar o maior acesso às vacinas para as crianças, sendo evidências robustas para subsidiar o gestor local e os profissionais de saúde no fortalecimento de estratégias de comunicação com a população sobre os riscos de doenças preveníveis por vacinas, possibilitando maior eficiência do PNI.

Recomenda-se o uso dos resultados do estudo como disparadores de processos de educação permanente em saúde, com intuito de estimular o diálogo a partir de discussões e reflexões da prática de vacinação, buscando as causas contextualizadas das baixas coberturas. Ademais, é necessário possibilitar a construção coletiva de estratégias locais para o alcance das metas estabelecidas pelo PNI, estimulando as possibilidades de instituir ações alternativas e inovadoras a partir da percepção dos profissionais de saúde e da comunidade.
